# Supplementary material for: All-at-once RNA folding with 3D motif prediction framed by evolutionary information
Source: Nat Methods. 2025 Oct 3;22(10):2094–106. doi: 10.1038/s41592-025-02833-w (PMC12510878; doi:10.1038/s41592-025-02833-w)
Supplement: Supplementary file 3 — Source code to run CaCoFold-R3D. Data and results. [file 41592_2025_2833_MOESM3_ESM.gz › supplemental_material/results/CaCoFold-R3D/CaCoFold-R3D_Rfam_vshuffle/RF00380_Magnesium.R2R.sto.pdf]

RF00380_Magnesium

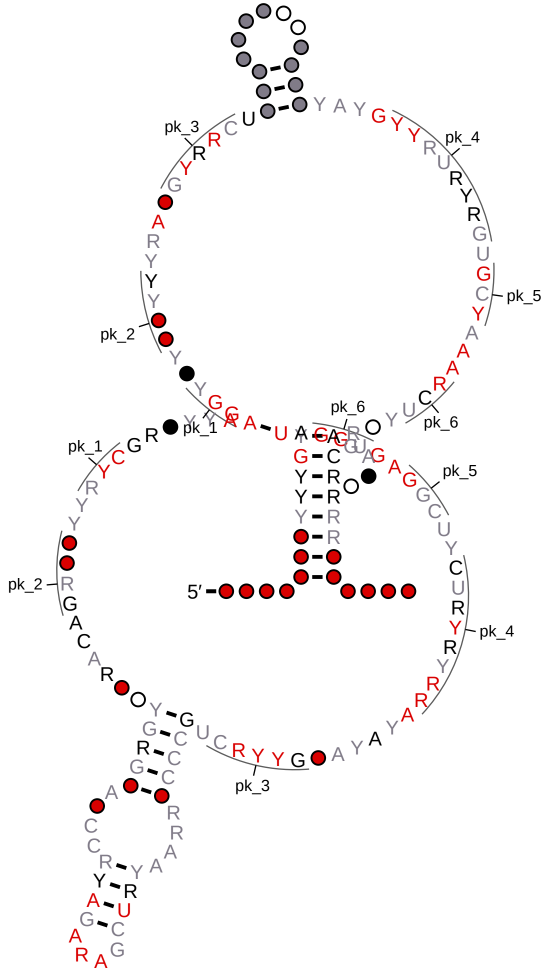

pk_1

R – Y
Y – G
C – G

pk_2

G – Y
R – Y

pk_3

G – C
Y – R
Y – R
R – Y
C – G

pk_4

C – G
U – R
R – Y
Y – R
R – U
Y – R
R – Y
R – Y

pk_5

G – Y
G – C
C – G

pk_6

G – U
G – C
U – R
